# Usability of a Fall Risk mHealth App for People With Multiple Sclerosis: Mixed Methods Study

**DOI:** 10.2196/25604

**Published:** 2021-03-22

**Authors:** Katherine Hsieh, Jason Fanning, Mikaela Frechette, Jacob Sosnoff

**Affiliations:** 1 Department of Kinesiology and Community Health University of Illinois at Urbana-Champaign Urbana, IL United States; 2 Department of Internal Medicine Section on Gerontology and Geriatric Medicine Wake Forest School of Medicine Winston-Salem, NC United States; 3 Department of Health and Exercise Science Wake Forest University Winston-Salem, NC United States; 4 Department of Physical Therapy and Rehabilitation Sciences School of Health Professions University of Kansas Medical Center Kansas City, KS United States

**Keywords:** smartphone, user center design, falls, mobile phone

## Abstract

**Background:**

Multiple sclerosis (MS) is a chronic, neurodegenerative disease that causes a range of motor, sensory, and cognitive symptoms. Due to these symptoms, people with MS are at a high risk for falls, fall-related injuries, and reductions in quality of life. There is no cure for MS, and managing symptoms and disease progression is important to maintain a high quality of life. Mobile health (mHealth) apps are commonly used by people with MS to help manage their health. However, there are limited health apps for people with MS designed to evaluate fall risk. A fall risk app can increase access to fall risk assessments and improve self-management. When designing mHealth apps, a user-centered approach is critical for improving use and adoption.

**Objective:**

The purpose of this study is to undergo a user-centered approach to test and refine the usability of the app through an iterative design process.

**Methods:**

The fall risk app Steady-MS is an extension of Steady, a fall risk app for older adults. Steady-MS consists of 2 components: a 25-item questionnaire about demographics and MS symptoms and 5 standing balance tasks. Data from the questionnaire and balance tasks were inputted into an algorithm to compute a fall risk score. Two iterations of semistructured interviews (n=5 participants per iteration) were performed to evaluate usability. People with MS used Steady-MS on a smartphone, thinking out loud. Interviews were recorded, transcribed, and developed into codes and themes. People with MS also completed the System Usability Scale.

**Results:**

A total of 3 themes were identified: intuitive navigation, efficiency of use, and perceived value. Overall, the participants found Steady-MS efficient to use and useful to learn their fall risk score. There were challenges related to cognitive overload during the balance tasks. Modifications were made, and after the second iteration, people with MS reported that the app was intuitive and efficient. Average System Usability Scale scores were 95.5 in both iterations, representing *excellent* usability.

**Conclusions:**

Steady-MS is the first mHealth app for people with MS to assess their overall risk of falling and is usable by a subset of people with MS. People with MS found Steady-MS to be usable and useful for understanding their risk of falling. When developing future mHealth apps for people with MS, it is important to prevent cognitive overload through simple and clear instructions and present scores that are understood and interpreted correctly through visuals and text. These findings underscore the importance of user-centered design and provide a foundation for the future development of tools to assess and prevent scalable falls for people with MS. Future steps include understanding the validity of the fall risk algorithm and evaluating the clinical utility of the app.

## Introduction

Multiple sclerosis (MS) is a chronic, neurodegenerative disease of the central nervous system (CNS) that affects over a million people in the United States [1]. MS may affect the brain, spinal cord, brainstem, and/or optic nerves and can result in a range of sensory (ie, pain and loss of proprioception), motor (ie, spasticity, muscle weakness, and balance or gait impairments), and/or cognitive (ie, slowed processing speed and memory loss) symptoms [2,3]. Symptoms vary on an individual basis, depending on which areas of the CNS are affected [2]. Furthermore, new symptoms may arise, or current symptoms may worsen throughout the course of the disease [4]. There is currently no cure for MS; however, treatments developed over the last two decades have slowed the disease progression and improved symptoms. Disease-modifying treatments, including injectable and oral drugs, have shown to be beneficial in ameliorating damage to the CNS, and trials using monoclonal antibodies and myelin restoration strategies suggest the potential for novel forms of MS therapy [5]. Although treatments have helped minimize the disease progression, the heterogeneity of MS makes this a complex disease to manage.

Mobile health (mHealth) apps have rapidly evolved in recent years to help individuals track, manage, and treat their health [6]. Due to the complexity of MS, there is increasing use of mHealth apps to support disease monitoring and symptom management [7,8]. More than 85% of people with MS own a mobile device, and 45% of people with MS use an mHealth app to help manage or treat MS [7]. The most common MS apps help with disease management or provide information about MS and MS treatment [9]. Other apps allow people with MS to connect with one another to share information and socialize, and others allow users to track their symptoms, mood, and energy over time [7].

Despite the number of MS-related apps, there are limited health apps developed to evaluate fall risk. Falls are a significant health concern for people with MS, with half of those falling in a 6-month period and up to 50% of falls resulting in an injury [4]. Current fall-related apps for aging and chronic disease populations focus on fall detection [10,11], whereas others measure movement tasks (ie, walking and sit to stand) as a proxy of fall risk [12-14]. Current fall-related apps, however, are not designed for people with MS who have unique risk factors and movement patterns compared with other chronic disease populations. In addition, they did not examine the multiple factors that cause falls in people with MS [4].

Risk factors for falls stem from multiple MS symptoms, including impaired walking and balance, cognitive decline, and fatigue [15]. Although fall risk assessments can be performed clinically, clinicians have time constraints, may not have the necessary equipment, and commonly only assess a single aspect of fall risk, usually asking for previous fall history [16]. Assessing fall risk, however, should include measuring multiple risk factors. Clinical fall risk assessments can include walking and balance tasks such as the Timed up and Go or Short Physical Performance Battery or falls self-efficacy and self-confidence questionnaires [17,18]. A fall risk app incorporating these tasks and measuring multiple risk factors can increase access to fall screening for people with MS and encourage the adoption of fall prevention strategies before a fall occurs. In addition, because MS symptoms fluctuate throughout the course of the disease [4], changes in symptoms lead to changes in fall risk. A fall risk app can help people with MS to measure and track these changes in their homes.

A fall risk mHealth app for people with MS offers access to fall assessment in the home setting, potentially improving fall risk self-management and reducing fall-related injuries. An mHealth app can measure fall risk by leveraging smartphone accelerometry to objectively measure postural control [19] and assess MS symptoms related to falls through self-reported questionnaires. A critical step in the development of an mHealth app is understanding the usability of the app for its intended users [20]. Usability testing ensures that those with MS can easily use and understand an app to improve their overall health. Moreover, a review of MS health apps indicated that most apps do not meet the needs of those with MS because they are not designed for the intended users, leading to poor adherence and use [9]. As people with MS have unique symptoms that may influence their technology use, applying a user-centered approach in the development of health apps can help improve their adoption and use [21]. Therefore, the purpose of this study is to develop a fall risk app for people with MS and to test the usability of the app through an iterative design process. A user-centered approach will improve the development of an app to facilitate the needs of those with MS to increase fall screening and ultimately reduce fall-related injuries [22].

## Methods

### App Development

**Figure 1:**
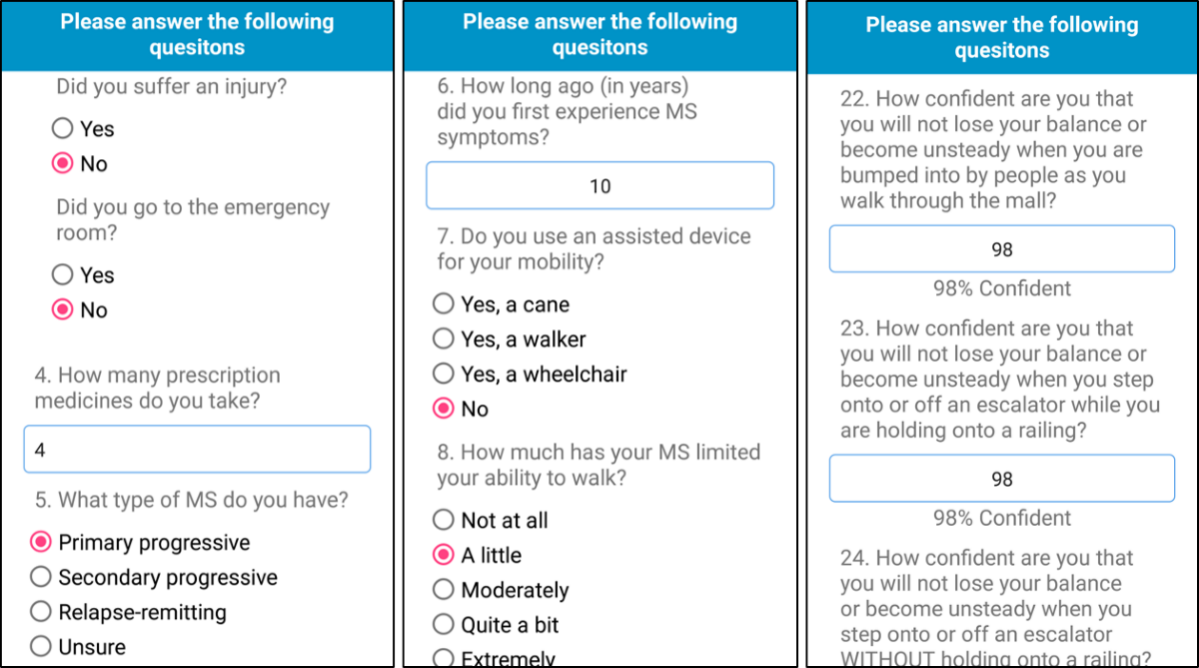
Screenshots of Steady-MS app asking users to answer 25 questions related to their health, past falls, multiple sclerosis symptoms, and perceived balance. MS: multiple sclerosis.

**Figure 2:**
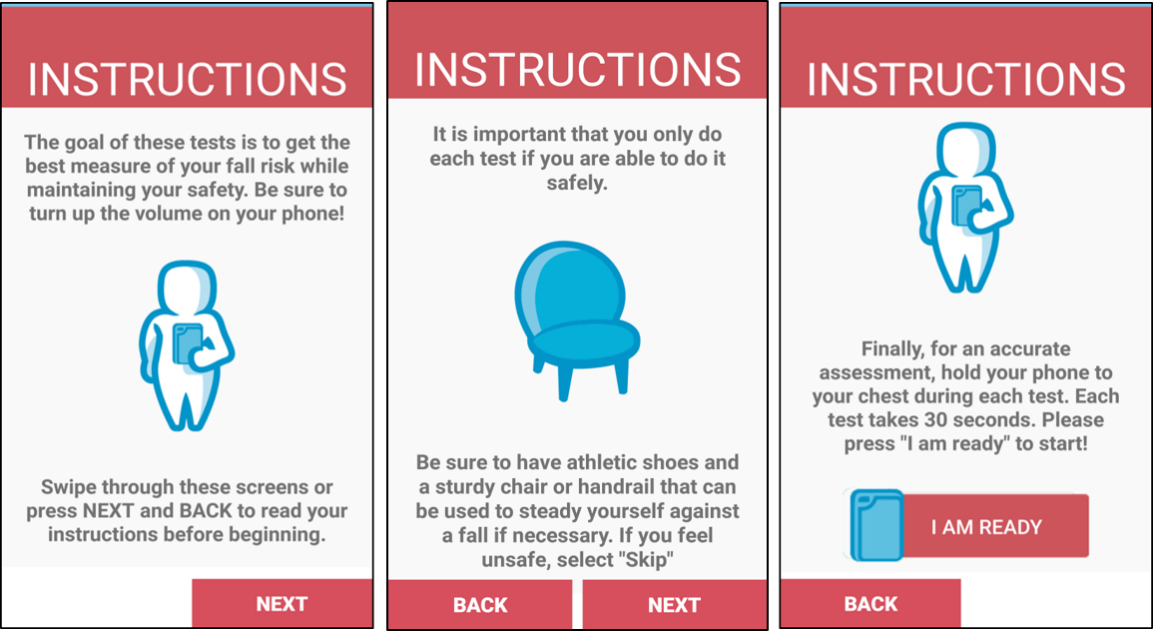
Screenshots of Steady-MS app guiding users to safely perform 5 standing balance tasks while holding the phone against their chest.

This app, Steady-MS, was developed in Android Studio 3.1.2 and was developed as an extension of a validated fall risk app for older adults, Steady [23]. Modifications were made to the questionnaire, balance tasks, and algorithm of Steady to apply specifically to the MS population. Steady-MS consists of 2 components: the first includes 25 questions targeting demographic information and MS symptoms (Multimedia Appendix 1). These questions include age, sex, past history of falls, type of MS, history of MS, the 12-item Multiple Sclerosis Walking Scale (MSWS-12) [24], and the short form of the Activities Balance Confidence Scale (ABC-6; Figure 1) [25]. These questions were specifically chosen because they are associated with falls in people with MS [17,26-28]. The second component, following the 25 questions, is a series of progressive balance tasks, in which the app guides users through 5 progressively difficult standing balance tasks. In the following order, the tasks are as follows: (1) eyes open, (2) eyes closed, (3) semitandem, (4) tandem, and (5) single leg. A text description and image guide users through each task (Figure 2). Each task takes 30 seconds, beginning with a 5-second countdown and the word *start* and ending with the word *stop*. The phone also vibrates at the start and end of each task. Users were instructed to hold the phone against their chest for the duration of the task to measure their postural sway. These tasks were chosen because worse performance on these tasks is associated with falls in people with MS [29,30]. After each task, users were asked to report if they (1) completed the task, (2) attempted but did not complete, or (3) did not attempt. Steady-MS measures postural sway by measuring acceleration in the mediolateral, anteroposterior, and vertical directions [19]. The Romberg ratio, the ratio between eyes open and closed, of the root mean square acceleration measured and recorded as the increased Romberg ratio, is associated with increased fall risk in people with MS [31]. The number of balance tasks completed, the root mean square Romberg ratio, and the responses to the 25-item questionnaire were inputted into a weighted algorithm and converted into a score ranging from 0 to 100, in which higher scores represent a higher risk for falls.

Steady-MS was also developed considering common MS symptoms that may influence usability. For instance, fatigue is a common symptom that affects approximately 70% of people with MS [32]. To prevent fatigue, we limited the total number of questions to 25 questions that were needed for the fall risk algorithm and asked only important additional questions (ie, MS duration and type of assisted device) that relate to falls. We also limited the balance tasks to 1 trial of the 5 tasks. Vision impairments are also an MS symptom affecting approximately 30% of people with MS and may influence reading questionnaires and instructions [33]. Therefore, the font size was at least 14, and we emphasized the high contrast between text and background. Cognitive impairment, including reduced processing speed and memory decline, affects between 40% and 70% of people with MS [34]. We aimed to prevent cognitive overload by presenting one set of instructions per screen and maintaining consistency throughout the app.

### Participants

A total of 10 people with MS participated in 2 usability rounds. It has been recommended that small groups (n=5) are suitable for identifying usability issues [35]. People with MS (n=5) interacted with Steady-MS and identified usability issues. Using their feedback, we improved the design of the app, and then, another group of people with MS (n=5) interacted with the app to identify any additional usability issues. This iterative design approach centered around the user is most effective for identifying user challenges when developing health apps [21,22]. Inclusion criteria for participants included (1) physician confirmed diagnosis of MS, (2) age 18 years or older, (3) self-reported ability to use a touchscreen device, and (4) ability to stand independently for at least 1 minute. Individuals with neurological disorders other than MS were excluded from the study. All procedures were approved by the Institutional Review Board, and all participants provided informed consent before participation.

### Procedures

An iterative design evaluation process of videotaped semistructured interviews was used to determine the optimal usability of Steady-MS. Participants were presented with a smartphone (Samsung Galaxy S6) and asked to open the app and follow all instructions as they completed both the in-app questionnaires and balance tasks. Participants first completed these steps independently, with as little assistance as possible. They then completed the in-app tasks a second time, but this time thinking aloud and narrating their thoughts. They were also encouraged to discuss their likes, dislikes, and recommendations for improvement. After receiving their fall risk score, participants were also asked to identify and draw different graphics of how they wanted to receive their score, such as on a circular chart or linear scale.

Following the semistructured interview, participants completed the Systematic Usability Scale (SUS) to understand the overall usability of the app. The SUS is widely used to quantify the usability of user-machine interfaces, consisting of 10 standard questions on a 5-point Likert scale [36]. The SUS ranges from 0 to 100, with higher scores representing greater usability. Previous work has indicated that the average technology SUS score is 60, and scores of 80 or above indicate that users are more likely to recommend the device to others [37]. Participants also completed the Mobile Device Proficiency Questionnaire (MDPQ) to understand their general proficiency in using mobile devices. The MDPQ ranges from 5 to 40, with higher scores representing greater technological proficiency [38]. Participants then completed the Expanded Disability Status Scale, a self-reported measure of disability that ranges from 0 to 10, with higher scores indicating greater disability [39].

After the first iterative cycle, changes were made to the app design based on the issues identified from the interviews. The second cycle of semistructured interviews was performed on 5 new participants with MS. Owing to COVID-19 restrictions on in-person research, interviews in the second round were performed remotely. The procedures followed the same format as the first round; however, participants were delivered a smartphone with Steady-MS installed, and interviews were conducted over a video call. This format allowed us to understand how Steady-MS is used in the home environment.

### Data and Statistical Analysis

All videotapes and field notes taken during the interviews were transcribed verbatim on a computer. Qualitative data from videotapes and field notes were analyzed to develop a coding system using MAXQDA (Version 12.3.3). On the basis of their content, data were assigned codes, and codes with similar content were grouped into themes. The codes and themes were reviewed and discussed by 2 researchers.

## Results

### Iteration 1

#### Overview

Participant characteristics are displayed in Table 1. From the semistructured interviews and coding analysis, 3 main themes were identified: (1) intuitive navigation, (2) efficiency of use, and (3) perceived value. Table 2 summarizes the main issues identified from the interviews and the subsequent changes made to Steady-MS.

**Table 1:** Demographic information of all participants in the first and second iterations.

Variables		Iteration 1	Iteration 2
Age (years), mean (SD)		53.2 (13.1)	54.6 (8.7)
**Gender, n (%)**			
	Female	4 (80)	3 (60)
	Male	1 (20)	2 (40)
EDSS^a^, median (IQR)		3 (2.5-6)	2.5 (2.5-6)
MS^b^ duration (years), mean (SD)		14 (5.9)	16.2 (9.2)
**MS type, n (%)**			
	Primary progressive	1 (20)	0 (0)
	Secondary progressive	0 (0)	1 (20)
	Relapse remitting	4 (80)	4 (80)
**Education, n (%)**			
	High-school diploma	0 (0)	1 (20)
	Associate’s degree	2 (40)	1 (20)
	Bachelor’s degree	3 (60)	1 (20)
	Master’s degree	0 (0)	2 (40)
**Mobile device use, n (%)**			
	Owns smartphone	5 (100)	4 (80)
	Owns tablet	2 (40)	3 (60)
Mobile device proficiency scale, mean (SD)		36.8 (3.3)	38.3 (1.1)

^a^EDSS: Expanded Disability Status Scale.

^b^MS: multiple sclerosis.

**Table 2:** Summary of the main issues identified in the first round of interviews, sample quotes from each issue, and solutions implemented to improve the app.

Domain and issue		Sample quotes	Solution
**Intuitive navigation**			
	Unclear if eyes are open or closed for balance tests	“I have to keep my eyes closed, don’t I?”	Added eyes to icons to depict if eyes are open or closed.
	Confusion between semitandem and tandem stances	“Maybe a picture or description because the one that said balance beam made more sense”	Modified pictures to clarify semitandem and tandem stances. Reworded description of each stance.
	Reentering ID before balance tests	“I just hit the *Get Started* again?”	After completing *About Me*, users are no longer prompted to reenter their ID.
	Redundant option of completing test	“I don’t understand *I did not attempt to complete the test* because if you didn’t attempt to complete it, why wouldn’t you just skip it?”	The *I did not attempt to complete the test* option was removed, as users are able to skip any balance task.
	Assisted device use	“This was to think about this as if I’m using my crutch, right?”	Added instructions to answer the activities balance confidence scale as if you were to have your assisted device.
**Efficiency of use**			
	Easy to use	“I find [Steady-MS^a^] easy to use on my own”	No solutions were needed.
**Perceived value**			
	Tracking score over time	If they can learn and improve their score, it would help them feel confident.	No solutions were needed.

^a^MS: multiple sclerosis.

#### Intuitive Navigation

The most common usability issue during the first iteration was related to intuitive navigation. When participants completed their self-reported questionnaires and moved onto the balance tasks, they were prompted to reenter their ID, a feature that was added to assist in testing several individuals simultaneously. Of the 5 participants, 2 had asked for clarification if they had to reenter their ID or if the app was finished. It was not intuitive for these participants to reenter their ID before moving onto the balance tasks. To address this issue, participants were no longer required to enter their ID to complete the balance tasks. In addition, participants who used an assistive device asked for clarification whether the questionnaires referred to using their assistive device or not. Therefore, for questions such as those from the short form of the ABC, we included instructions regarding assisted device use.

**Figure 3:**
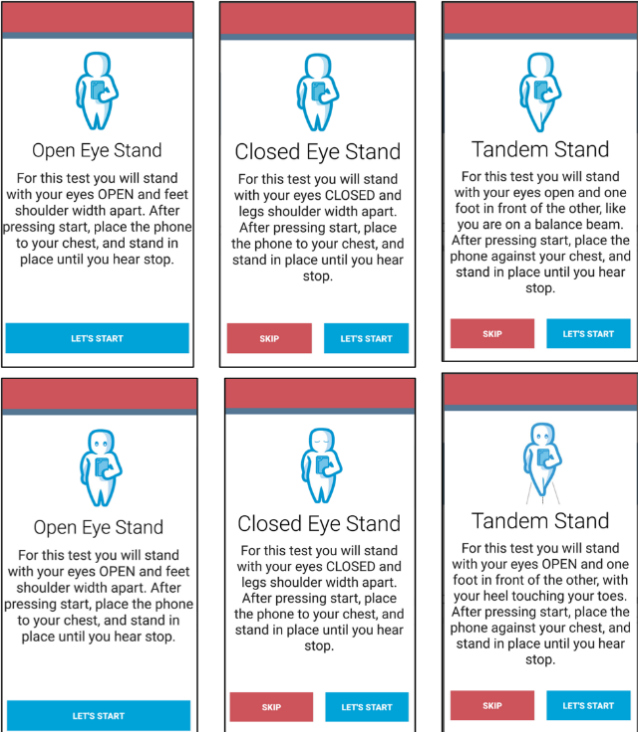
Screenshots of Steady-MS app guiding users through progressive balance tasks. The top panel of screenshots depict the first iteration of images and text, and the bottom panel depicts the second iteration of images and text. Images of eyes and rewording of text were edited to improve clarity and reduce cognitive overload.

There was also difficulty in navigating through the 5 balance tests. Two of the participants asked for clarification if their eyes were open or closed, whereas 2 different participants performed the semitandem and tandem conditions incorrectly based on observation and video recording from the research staff (Table 3). Although there was a text description instructing each balance stance, these participants reported that they preferred to have a clearer image rather than reading text. In addition, following each balance task, participants were asked to rate if they completed each test with 1 of the 3 options. Of these options, participants reported that the last option, “I did not attempt to complete the test,” was not intuitive. Participants indicated that if they were to select this option, they would have chosen to skip the test. To address these issues, we modified the images to indicate that the eyes should be open or closed for each task (Figure 3). We also eliminated the option “I did not attempt to complete the test.”

**Figure 4:**
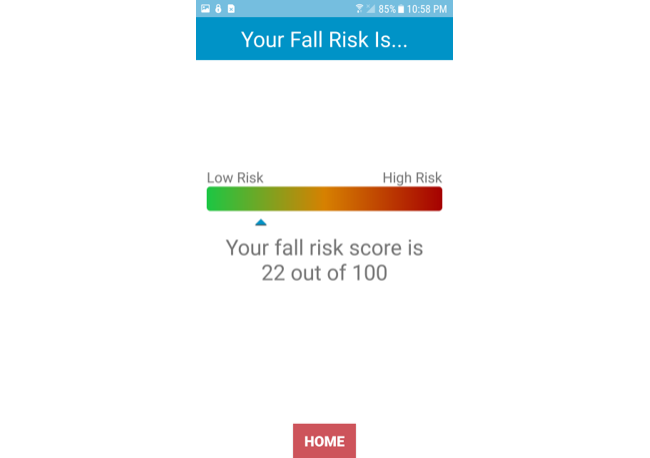
After completing the balance tests, Steady-MS app outputs an overall fall risk score ranging from 0 to 100, with higher scores representing a higher risk of fall.

For the final fall risk score, participants also reported that they liked receiving an overall score; however, using a scale to present their score would be the most intuitive to improve their understanding. A total of 3 participants preferred using a horizontal or vertical scale, as opposed to a circular chart. They reported that they understood their score better on a linear scale with *low risk* on one end and *high risk* at the opposite end. Therefore, we added a horizontal scale depicting the user’s final fall risk score (Figure 4). The score ranges from 0 to 100, with a green color corresponding to lower fall risk and a red color corresponding to higher fall risk:

I’m a visual person, and when I have to read something, I will default to looking at the picture. I mean, I can read an instruction manual all day and, but if you show me a picture or video on how to do it, I’ll probably pick it up faster.

Participant, male, 36 years old

You know, like they do on emojis. You just have those little circles for your eyes if they are closed or open. Maybe it’s just me, but it’s reading all these words or looking at the picture. I could see what I was supposed to do without reading all that.

Participant, female, 57 years old

I don’t understand ‘I did not attempt to complete the test’ because if you didn’t attempt to complete it, why wouldn’t you just skip it?

Participant, female, 46 years old

I enter my ID again and hit the ‘Get Started’?

Participant, female, 76 years old

**Table 3:** Description, order, and number of participants who correctly performed each of the 5 balance tasks in Steady—multiple sclerosis.

Task order	1	2	3	4	5
Visual task	Eyes open	Eyes closed	Eyes open	Eyes open	Eyes open
Feet position	Shoulder width apart	Shoulder width apart	Semi tandem	Tandem	Single leg
Iteration 1 correct performance, n (%)	5 (100)	5 (100)	3 (60)	3 (60)	5 (100)
Iteration 2 correct performance, n (%)	5 (100)	5 (100)	4 (80)	5 (100)	5 (100)

#### Efficiency of Use

Overall, all participants found that Steady-MS was efficient and easy to use. Participants reported that the app walks them through each question and balance test and that they could use it independently. One participant reported that the MSWS-12 questionnaire felt redundant; however, none of the participants felt that the total number of questions or number of balance tasks needed to be reduced:

I mean, it is pretty easy and seems to walk you through it, in my opinion. It’s pretty straight forward.

Participant, male, 36 years old

Everything was quite clear when I was going through that.

Participant, female, 51 years old

I could do that on my own.

Participant, female, 57 years old

#### Perceived Value

The last theme was related to the value of having a fall risk app. All participants reported that having an app would be beneficial for them to understand their risk of falling. Two of the participants found that having a fall risk score can provide confirmation or reassurance in their perceived changes in symptoms, especially during a relapse. These participants said that they would want to use Steady-MS to gauge their changes before seeing a physician. Participants with a higher fall risk found value in learning about their scores; however, they also wanted exercises or other prevention strategies. One participant also reported that she sees value in monitoring her fall risk at home rather than having to travel to a clinician.

Other participants reported that going through the app helped them realize the factors related to falls. One participant, for instance, learned the importance of vision for fall risk and could be more aware of this in the future. Another participant reported that the tandem stance was a balance task that she wanted to improve on:

I guess I didn’t realize the factors if your eyes open or closed or your stance can increase your fall risk. I guess I can be more conscious about those types of things because it seems to me now with that feedback about my vision, it plays a pretty important role in my balance.

Participant, female, 57 years old

But when I get feeling bad, boy, that number [the fall risk score] shoots up. You know? It’s not just my mind, you know, the app kind of confirms it. So maybe I’ll use a cane instead.

Participant, male, 36 years old

I like to gauge without having to go all the way to the doctor.

Participant, female, 46 years old

### Iteration 2

After the second round of interviews, transcript analysis and coding revealed 3 themes related to intuitive navigation, efficiency of use, and perceived value.

#### Intuitive Navigation

**Figure 5:**
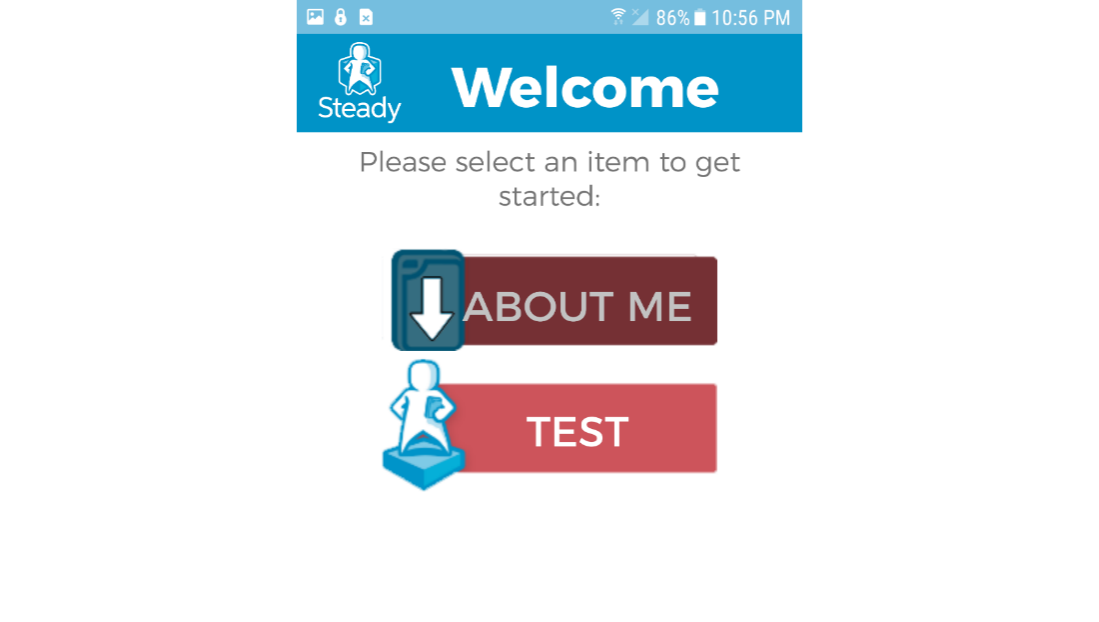
After completing the "About Me," this section is dimmed and users are prompted to click on the "Test" section.

After modifying Steady-MS, participants in the second round of interviews reported little difficulty navigating through the app. After editing the images and text for the balance tasks, 4 of the 5 participants performed all of the balance tasks correctly based on observation and video analysis. One participant asked for clarification of the semitandem stance to confirm if she was standing correctly. After completing the *About Me* questions, 1 participant returned to the questionnaires again, realized that there were no additional questions, and proceeded to complete the balance questions. To indicate that this section is completed, we dimmed the *About Me* section after users finished the questions (Figure 5). Overall, the participants reported that Steady-MS was intuitive and easy to navigate:

I didn’t know if there was more about me, like if there were more categories within it. So I chose it again and then I kind of knew enough to be able to scroll through and go back.

Participant, female, 56 years old

[Referring to the fall risk scale] The green and the red colors [were] pretty self-explanatory to me.

Participant, female, 53 years old

#### Efficiency of Use

Similar to the first round of interviews, participants reported that Steady-MS was efficient and simple to use. They found that navigating through the questions and balance tasks was straightforward. Participants reported that if there was any confusion on the balance tasks or questionnaires, they understood the instructions after rereading a second time. The participants also reported that they could use Steady-MS independently without additional guidance:

It seems simple enough to use and I’m not tech savvy as some are. There wasn’t anything if I read through it twice I wouldn’t understand.

Participant, male, 61 years old

It’s very easy to read. I liked that part, and the contrast is good too. I’m actually reading without my reading glasses, so that’s a good sign.

Participant, female, 53 years old

I thought it was pretty good and straightforward.

Participant, male, 64 years old

#### Perceived Value

All participants reported that Steady-MS can provide many benefits. Participants indicated that the most beneficial component was seeing their fall risk score. For instance, one participant said that when she sees her neurologist, she may be asked to perform static balance tasks but does not receive feedback on her performance. With Steady-MS, she can see a score that gives her measurable feedback. Another participant reported that Steady-MS may be useful in understanding her changes in fall risk with lifestyle changes. Due to COVID-19, her yoga classes have been canceled. She can feel changes in her balance and walking as a result; however, seeing a score to confirm these changes may motivate her to try online yoga:

I think it’s neat to gauge your risk. Like when I go to the neurologist, she’ll have me do stuff, and she’ll say hmm or uh huh. And I don’t know what any of that means. So it’s kind of nice to have it be like, oh, your score is this.

Participant, female, 39 years old

They’re doing a lot of yoga online and whatnot. But we all know we don’t do those, or I don’t anyway, as much as I would if I were going to class. So it might be a way for me to say, hey, you need to do a little bit more with your yoga because your balances are getting a little bit more, you know, unstable, I suppose.

Participant, female, 43 years old

You live with yourself all day, every day, and sometimes it’s hard to tell if you feel like, you know, like I’m not getting around as well. And if you could look at [the app] and would it show you, oh yeah, it does say I have more of a fall risk.

Participant, female, 56 years old

### System Usability Scale

In the first iteration, the average SUS score was 95.5, with a standard deviation of 3.3. In the second iteration, the average SUS score was 95.5, with a standard deviation of 2.9. Although the SUS score did not change between iterations, this is likely because of a ceiling effect with a maximum score of 100. These high scores suggest that participants are likely to recommend Steady-MS to others [37].

## Discussion

### Principal Findings

The purpose of this study is to test and refine the usability of a fall risk health app for individuals with MS through a user-centered design approach. After the first round of usability testing, participants identified issues navigating through the app but reported that it was easy to use and found value in undergoing a fall risk assessment. We modified the app to improve navigation, and after the second round of testing, participants reported that the app was easy to navigate and could use the app on their own. These results, complemented with high SUS scores, suggest that Steady-MS is a usable health app that people with MS can use to self-assess their risk of falling.

Importantly, our results underscore the need for a user-centered design during the development of health apps. Indeed, the main issues identified from semistructured interviews were related to intuitive navigation, and a health app with poor navigation is unlikely to be used. These issues were related to understanding the entire instructions of a balance task (ie, the position of the feet and if eyes are open or closed). Cognitive impairment is a common symptom in people with MS [40], and the instructions for each balance task may result in cognitive overload in people with MS. To reduce cognitive overload, we improved the visuals and text to depict each balance task. Indeed, during the second round of testing, 4 participants completed all balance tasks correctly without asking for clarification. For future developments, it is important to consider the cognitive demands of people with MS to prevent cognitive overload.

Participants in both rounds of testing reported that they found the app clear, simple to use, and useful in learning their risk of falling. This suggests that people with MS can independently use carefully designed health apps such as Steady-MS and learn about their fall risk. Participants also reported that they value receiving a fall risk score because they can identify whether their score improves with exercise or declines with the onset of symptoms. Steady-MS offers the potential for people with MS to self-assess and self-monitor their fall risk using a smartphone. As MS symptoms fluctuate throughout the course of the disease, their risk of falls also changes [4]. Therefore, tracking and monitoring fall risk can help people with MS increase their awareness of their fall risk and take part in prevention strategies before a fall occurs. Unlike traditional fall risk assessments performed in clinics or laboratory-based settings, Steady-MS provides a tool to increase access to fall risk assessments that can be performed at home.

### Lessons Learned

When developing future mHealth apps for people with MS, there are important aspects to ensure high usability. First, it is important to prevent cognitive overload in people with MS, as cognitive impairment is a common symptom of MS [40]. Within Steady-MS, cognitive impairment was found when participants were asked to follow 2 separate instructions for a balance task. Using clear visuals and simple text is important to avoid cognitive overload. Second, when presenting a score or number to people with MS, it is important to ensure that the score is easily understandable. Participants in the study reported that they preferred receiving a number because it was measurable, and they could track improvements over time. However, it is important that people with MS accurately interpret scores. When using a scale from 0 to 100, it was important to depict, both visually and through text, that 0 represents low risk and 100 represents high risk. These 2 guidelines can improve the development of future health apps to maximize the usability of people with MS. Third, using a user-centered, iterative approach in designing Steady-MS resulted in users effectively and efficiently understanding new instructions. This approach may also apply to other clinical populations with physical and cognitive impairments when designing a health app.

### Limitations

This is the first study to develop and test an MS fall risk mHealth app; however, there are also limitations to this study. The participants in this study had high mobile technology use and scored high on the MDPQ. Those with less technology experience may have additional usability issues that were not identified in this study. However, MS commonly affects younger adults, and more than 80% of people with MS own a smartphone [7]. Therefore, it is likely that a person with MS will already have mobile device experience. Additionally, while Steady-MS measures overall fall risk, it currently does not offer fall prevention strategies. Seeking treatment after understanding individual risk is the next step to prevent falls, and future steps should aim to include tailored fall prevention strategies and understand if people with MS adopt these strategies. Future steps should also understand the validity of the algorithm and display the results of individual components that contribute to fall risk. This may help guide people with MS with specific fall prevention strategies. Saving fall risk scores may also help people with MS monitor their changes over time. In addition, future work should aim to understand how fall risk apps such as Steady-MS can be incorporated into clinical care. Although Steady-MS was designed for use at home, integrating fall risk apps with clinical guidance in a safe manner can increase access to fall prevention strategies. This study of 10 participants is also a small sample size, and future steps should include a larger, diverse sample to understand usability needs across the heterogeneous MS population. Finally, although participants reported high perceived value in learning about their fall risk score and offered suggestions to improve how the displaying the score, future interviews, and studies should understand how to present individualized fall risk information to prevent negative, unintended consequences.

### Conclusions

In conclusion, the purpose of this study is to determine the usability of a fall risk health app for people with MS. After one round of semistructured interviews, we made modifications to improve users’ intuitive navigation when answering their health-related questionnaires and performing 5 balance tasks. After a second round of interviews, users reported that the app was straightforward to use and easy to navigate and that they found value in learning about their fall risk. SUS scores averaged 95.5 after the second round of testing, suggesting high usability. These results supported the use of a fall risk app to provide people with MS a tool to self-assess and self-manage their fall risk. Moreover, these results underscored the importance of using a user-centered design approach to identify usability challenges when developing mobile apps for individuals with chronic diseases.

## Appendix

Multimedia Appendix 1 Steady-MS “About Me” questions.

## Abbreviations

ABC: Activities Balance Confidence Scale
CNS: central nervous system
MDPQ: Mobile Device Proficiency Questionnaire
mHealth: mobile health
MS: multiple sclerosis
MSWS-12: The 12-item Multiple Sclerosis Walking Scale
SUS: System Usability Scale
