# Supplementary material for: Usability of a Fall Risk mHealth App for People With Multiple Sclerosis: Mixed Methods Study
Source: JMIR Hum Factors. 2021 Mar 22;8(1):e25604. doi: 10.2196/25604 (PMC8080269; doi:10.2196/25604)
Supplement: Multimedia Appendix 1 [file humanfactors_v8i1e25604_app1.docx]

Supplementary Material: Steady-MS About Me Questions

1. What is your current age in years?
2. What is your gender?
3. Have you fallen in the last year?
   1. If yes: How many times?
   2. If yes: Did you suffer an injury?
   3. If yes: Did you go to the emergency room?
4. How many prescription medications do you take?
5. What type of MS do you have?
6. How long ago did you first experience MS symptoms?
7. Do you use an assisted device as your primary form of mobility?

Questions 8-19 ask how MS has affected your walking in the last 2 weeks

1. How much has MS limited your ability to walk?
2. How much has MS limited your ability to run?
3. How much has MS limited your ability to climb up and down stairs?
4. How much has MS made standing when doing things more difficult?
5. How much has MS limited your balance when standing or walking?
6. How much has MS limited how far you can walk?
7. How much has MS increased the effort needed for you to walk?
8. How much has MS made it necessary for you to use support when walking indoors?
9. How much has MS made it necessary for you to use support when walking outdoors?
10. How much has your MS slowed down your walking?
11. How much has your MS affected how smoothly you walk?
12. How much has your MS made you concentrate on your walking?

If you use a walking aid, please rate the following questions is if you are using those supports.

1. On a scale from 0-100%, how confidence are you that you will not lose your balance or become unsteady when you stand on your tiptoes and reach for something about your head?
2. On a scale from 0-100%, how confidence are you that you will not lose your balance or become unsteady when you stand on a chair and reach for something?
3. On a scale from 0-100%, how confidence are you that you will not lose your balance or become unsteady when you are bumped into people as you walk through the mall?
4. On a scale from 0-100%, how confidence are you that you will not lose your balance or become unsteady when you step onto or off an escalator while you are holding onto a railing?
5. On a scale from 0-100%, how confidence are you that you will not lose your balance or become unsteady when you step onto or off an escalator WITHOUT holding onto a railing?
6. On a scale from 0-100%, how confidence are you that you will not lose your balance or become unsteady when you walk outside on icy sidewalks?
